# Daily prosocial actions during the COVID-19 pandemic contribute to giving behavior in adolescence

**DOI:** 10.1038/s41598-022-11421-3

**Published:** 2022-05-06

**Authors:** Sophie W. Sweijen, Suzanne van de Groep, Kayla H. Green, Lysanne W. te Brinke, Moniek Buijzen, Rebecca N. H. de Leeuw, Eveline A. Crone

**Affiliations:** 1 Erasmus School of Social and Behavioural Sciences, Erasmus University Rotterdam, Burgemeester Oudlaan 50, 3062 PA Rotterdam, The Netherlands; 2 Radboud University Nijmegen, Nijmegen, The Netherlands

**Keywords:** Psychology, Human behaviour

## Abstract

Prosocial actions are a building block for developing mature and caring social relations. However, the global pandemic may hamper adolescents’ prosocial actions. In this preregistered study, we examined the extent to which adolescents provided daily emotional support during the COVID-19 pandemic. In total, 10–25-year-old high school and university students participated at three timepoints (*N* = 888 at the first timepoint (May 2020); 494 at the second timepoint (Nov 2020) and 373 at the third timepoint (May 2021)). At the first and second timepoint, participants completed 2 weeks of daily diaries on providing emotional support. At all timepoints, participants performed Dictator Games to measure giving to peers, friends and COVID-19 targets (medical doctors, COVID-19 patients, individuals with a poor immune system). Across the three timepoints, adolescents gave more to COVID-19 targets than peers and friends, but giving to COVID-19 target was highest in the beginning of the pandemic (first timepoint relative to second and third timepoint). Results from the first timepoint showed that emotional support directed to friends peaked in mid-adolescence, whereas emotional support towards family members showed a gradual increase from childhood to young adulthood. Furthermore, daily emotional support increased between the first and second timepoint. Daily emotional support to friends predicted giving behavior to all targets, whereas emotional support to family was specifically associated with giving to COVID-19 targets. These findings elucidate the relation between daily actions and prosocial giving to societally-relevant targets in times of crisis, underlying the importance of prosocial experiences during adolescence.

## Introduction

The COVID-19 pandemic puts a large burden on the younger generation, who suffer from feelings of loneliness and anxiety^1,2^. The limited to no physical contact with peers and being constrained to stay at home is expected to specifically affect adolescents^3^, who transition from childhood to adulthood. This transition is marked by a significant expansion of their social environment^4^. Adolescence is especially important for adapting to one’s social environment^5^, which eventually enables individuals to take on a mature role within society. The pandemic may affect not only mental health, but also opportunities for daily social experiences, such as providing support, sharing, and giving^2,6^.

The restrictions in social experiences during the COVID-19 pandemic may hamper well-being of a young generation during their formative years^7^. Adolescence is a period in life typified by strong needs for exploration, forming new social relationships and rapid adjustment to changing social contexts^5^. Therefore, limiting social experiences may hamper the possibility to engage in meaningful social interactions, which are important for societally participatory behaviors^8,9^ as well as for the mental well-being of adolescents^10^. At the same time, studies on the immediate effects of the pandemic lockdown showed a larger willingness of individuals to contribute to community benefits and helping behavior^6,11^. The few studies that examined the effects of the pandemic on social behavior and associated care for well-being of others during adolescence show substantial heterogeneity among individuals^12^. In this preregistered study with a large dataset of individuals in adolescent development (10–25-years, *N* = 888 in May 2020, *N* = 494 in November 2020, and *N* = 373 in May 2021), we therefore aim to test how providing daily emotional support to friends and family is associated with experimental giving to others in need, and how this association is moderated by emotional and cognitive factors.

### Prosocial behavior

Prosocial behavior, defined as voluntary behavior to benefit others, such as helping and comforting, shows a peak in mid-adolescence to friends^13^, although the developmental pattern depends on social context^14^. Prosocial actions have various benefits for the benefactor, particularly during adolescence as a developmental period of navigating through more complex social worlds^4^. This highlights the importance of prosocial experiences in a developmental life phase characterized by spending more time with peers and developing more egalitarian relationships with caregivers^15^. Prior studies show a larger reward drive in adolescence^16^, which is associated with risky behaviors that potentially have detrimental health consequences for self and others, such as alcohol use and reckless behavior^17^. However, recent accounts have demonstrated that this same reward drive may also underlie tendencies to help others^13^. In addition to supporting friends, providing assistance to family has previously also been associated with a larger reward drive^18^. Thus, the same neural and behavioral reward sensitivity may underlie both risk seeking and seeking opportunities for prosocial actions^19^. Initial results on the effects of the first weeks of lockdown demonstrated a decrease in opportunities for prosocial actions, such as providing emotional support to friends^6^. However, no evidence is available on how this behavior changes during the pandemic, whereas this is a time where there are significant restrictions in the possibility to spend time with others. Therefore, an urgent question concerns the opportunities that adolescents have to provide emotional support towards friends and family during the pandemic, the daily frequency of prosocial actions, and their effects on other types of prosocial actions, such as giving.

Prosocial actions can be directed to friends and family, but also to unknown others such as individuals who are deserving or in need^15^. In prior research, it was found that adolescents give more to friends but less to unknown others and that this differentiation increases during adolescence^20^, possibly indicating an emerging ingroup-outgroup distinction^21^. A recently developed Pandemic Dictator Giving paradigm allowed us to examine giving behavior towards COVID-19 targets^6^. Targets of giving were friends, unknown others, or targets that were in need during the COVID-19 pandemic. In the current study, we investigated adolescents’ giving to different targets in the Pandemic Dictator Game at three timepoints during the pandemic to examine how giving changes over time. We further examined how experimental giving was associated with age, and to what extent daily emotional support was predictive of experimental giving.

### Individual differences in cognitive and emotional tendencies

Prior research has established that individual differences in benefactor personal tendencies influence the extent to which adolescents display prosocial behaviors^15^. Emotional and cognitive factors have previously been associated with prosocial behaviors, suggesting potential moderating effects of these factors. First, experiencing social reward from prosocial actions may impact adolescents’ subsequent prosocial behavior^14^. Second, executive functions are cognitive factors that may moderate strategic prosocial behavior^18,22^. Third, the willingness to contribute to society may impact adolescents’ prosocial behaviors, because this willingness may reflect adolescents’ other-oriented motives^23,24^. Finally, altruistic behavior was previously found to correlate with giving and sharing^24^. We therefore examined whether these four emotional and cognitive factors (i.e., experiencing social reward from prosocial actions, executive functions, the willingness to contribute to society, and altruistic behavior) are individual difference factors that may moderate the relation between prosocial actions and prosocial outcomes.

### The present study

In this preregistered longitudinal daily diary study during the COVID-19 lockdown in May and November 2020 with a follow-up study in May 2021, we examined prosocial behavior during the pandemic among adolescents aged 10 to 25 years (*N* = 888 at first timepoint, *N* = 494 at second timepoint, *N* = 373 at third timepoint). The central aim of the present study was to examine daily emotional support towards friends and family^6,25^, and the extent to which this was associated with experimental giving during the COVID-19 pandemic, with potential moderating influences of emotional and cognitive factors. Based on previous studies demonstrating gender differences and nonlinear developmental trajectories^13^, we also exploratory investigated the effects of linear and quadratic age and gender on daily opportunities for prosocial actions and experimental giving behavior. See Supplementary S1 for more details on the preregistration.

First, we exploratorily examined whether adolescents would show differences in daily emotional support to friends and family, following up on research that shows the relative importance of friends in adolescence^6,26,27^. Second, according to our preregistered hypotheses we expected that adolescents would show a decrease in emotional support over the course of the pandemic between May 2020, November 2020 and May 2021^6^. Third, we hypothesized in our preregistration that in the Pandemic Dictator Game, on average, adolescents would give the least to strangers, more to friends, and most to people in need (i.e., doctors at a hospital, COVID-19 patients, or individuals with a poor immune system)^6^. Fourth, we expected that adolescents would show a decrease in giving over the course of the pandemic^6^. We exploratory examined whether adolescents would show changes in giving behavior towards the different targets in the Dictator Game, based on a recent study on the first weeks of lockdown demonstrating that adolescents show differences in giving towards different targets^6^. Fifth, based on the assumption that prosocial actions can be experienced as rewarding and can therefore reinforce future prosocial behaviors, in our preregistration we expected that adolescents who provided more daily emotional support were more likely to give to others in the Pandemic Dictator Game^19^. Here, we formulated different expectations for the separate targets in the Dictator Game, that is: we expected a general positive relation between prosocial actions and giving (main effect) and we expected that these effects would be larger for deserving targets or targets in need (target interaction). Sixth, as put forward in our preregistered hypotheses, we expected that how much adolescents give in general, and the extent to which they differentiate between targets, would be moderated, and specifically, positively affected, by emotional and cognitive factors, specifically: sensitivity to prosocial rewards, executive functions, general contributions to society, and altruism^14,15,18,22–24^. Here, we also examined the moderating effects of emotion awareness and self-control on this relation based on previous studies demonstrating associations between these factors and prosocial behaviors^22,28^. Even though the moderator analyses for emotion awareness and self-control were preregistered as main analyses, the hypotheses and results of these specific analyses can be found in Supplementary S2 for brevity and clarity of the present study. Finally, all analyses included socioeconomic status (SES) as control variable, because recent studies have shown that SES may impact prosocial behavior, also within the context of the Dictator Game^29^.

## Methods

### Participants

**Figure 1:**
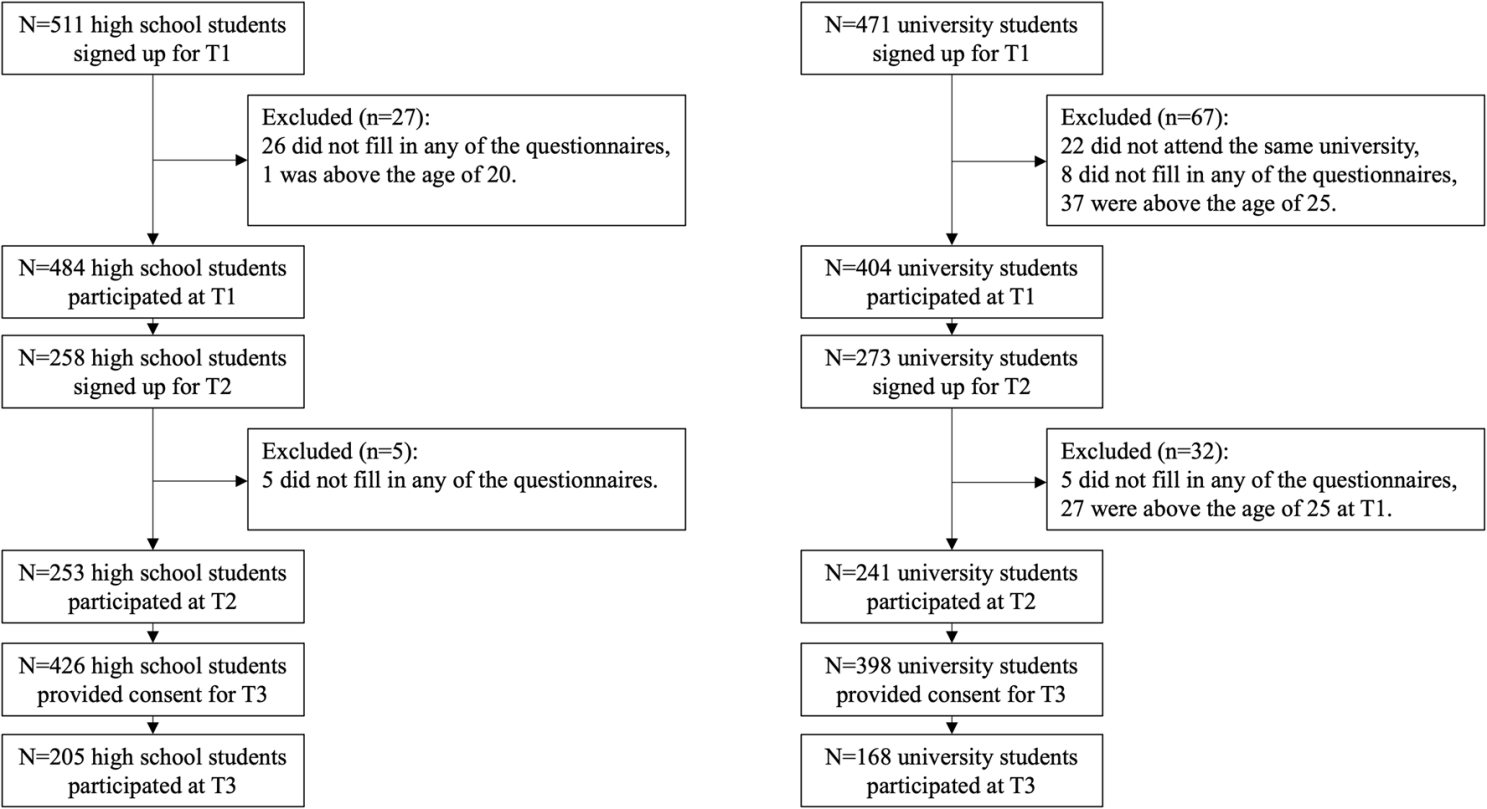
Flowchart of participants.

Two adolescent samples participated in the current study: a high school student sample and a university student sample. High school students were recruited through Dutch high schools in the Rotterdam area in the Netherlands. University students attended a program at Erasmus University Rotterdam (EUR) and were approached through the university website, email and social media platforms. See Fig. 1 for a detailed flowchart of the inclusion and exclusion of participants according to our preregistered criteria (see https://osf.io/h5x2a/). The first timepoint in May 2020 (T1) included a final sample of 888 adolescents, consisting of a sample of 484 high school students (*M* age = 15.31, *SD* = 1.78, age range 10–19, 63% females) and 404 university students (*M* age = 21.48, *SD* = 1.91, age range 17–25, 81% females). The majority of the final sample was of Dutch ethnicity (80%) and had middle to high SES based on parental educational level (low-middle-high = 6%-24%-63%). For the second timepoint in November 2020 (T2), the final sample included 494 adolescents, with 253 high school students (*M* age = 16.02, *SD* = 1.79, age range 11–19, 76% females) and 241 university students (*M* age = 21.83, *SD* = 1.88, age range 18–26, 84% females). For the third timepoint in May 2021 (T3), 205 high school students (*M* age = 16.37, *SD* = 1.79, age range 11–20, 64% females) and 168 university students (*M* age = 22.53, *SD* = 1.88, age range 18–26, 81% females) participated at T3, resulting in a total sample of 373 adolescents. Given the differences in sample size between the timepoints, we first performed all analyses for the first timepoint separately to include the largest sample as possible, consistent with the preregistration (i.e., we tested for replications of the results of T1 in the sample at T2), and then reported the longitudinal comparisons of participants who participated at multiple timepoints.

To examine whether there were significant differences between participants who partook at multiple timepoints from those who only partook at T1, the samples of participants with complete data (i.e., three timepoints) and of participants with incomplete data (i.e., only T1) were compared on demographic variables (i.e., age, gender, and SES). The samples of participants showed no differences regarding age and SES (both *p*’s > 0.05). However, the sample of participants who participated at all three timepoints consisted of more females (68%) compared to the sample of participants who partook only at T1 (62%), χ^2^ (1) = 25.61, *p* < 0.001.

### Procedure

**Figure 2:**
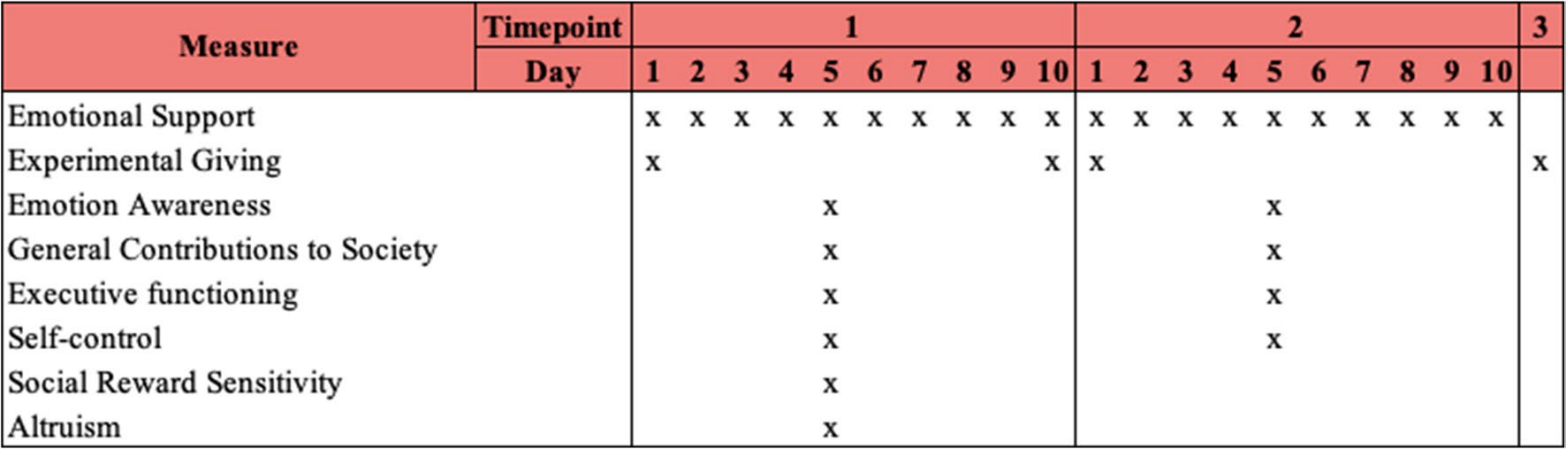
Timeline of administered measures at each timepoint.

For the first two timepoints (T1 and T2), data were collected through online daily questionnaires via the Qualtrics domain on weekdays during a period of two consecutive weeks (i.e., 10 daily measurements) at both timepoints. Each questionnaire consisted of daily measures assessing, amongst others, daily actions in providing emotional support to friends and family during the COVID-19 pandemic (duration 5–10 min). On testing day 5 (T1 and T2), a set of additional measures was administered to assess individual differences in social reward sensitivity, executive functioning, altruism, and general willingness to contribute to society (duration 15–20 min). Finally, on the first and final day of T1 and T2 a set of additional measures was obtained to measure experimental giving behavior (duration 15–20 min). At T3, all measures were included in one single questionnaire. See Fig. 2 for an overview of all administered measures at each timepoint. Additional questionnaires were administered as part of a larger study but are beyond the scope of the current article. See our OSF-page (https://osf.io/h5x2a/) for an overview of all measures and the preregistered steps in the data preparation. Whereas all high school students filled in the questionnaires in Dutch, university students were given the choice to fill in the questionnaires either in Dutch or in English, depending on their preferred language. See Supplementary S3 for additional information on the study procedure.

This study was reviewed and approved by the Ethics Committee of the Erasmus School of Social and Behavioral Sciences of the Erasmus University Rotterdam and was performed in accordance with the guidelines and regulations of the ethics committee. Informed consent was provided by participants and, for minors (16−), also by parents. Participants received 15 euros for participating at the first two timepoints (T1 and T2), regardless of the number of daily questionnaires they completed. At the follow-up study T3, participants received 10 euros.

Given that this study concerns the impact of the COVID-19 pandemic, we present here the specific COVID-19 related governmental restrictions in the Netherlands between May 2020 and May 2021. During the first two timepoints (May and November 2020), for individuals aged 13 years or older, restrictions were social distancing to adults (1.5 m physical distance), no gatherings outside the family context, limited visitors at home, staying home in case of symptoms, and closing of high schools and universities. In May 2021, limited gatherings outside the family context were allowed (no more than 2 people per day), and high schools and universities were partially open.

### Prosocial measures

#### Daily emotional support

The Emotional Support subscale of the Opportunities for Prosocial Actions (OPA) was administered to assess daily emotional support during the COVID-19 pandemic^6^. Here, we adapted the original version to include two targets. That is, we assessed the extent to which participants emotionally supported their friends and family members on a daily basis, using a 5-point scale with a range from 0 (not at all) to 5 (a lot). The questionnaire consisted of three items per target: both for friends and family members. These items were as followed: (1) ‘I comforted friends/family members when they were upset’, (2) ‘I sent a message to friends/family members (and/or called them), to be kind’, and (3) ‘I did my best spend time with friends/family members’. Here, we provided no specific instructions on how support can take many different forms (e.g., online versus offline). Prosocial opportunities can vary widely on a daily basis. Therefore, the OPA was administered on all ten testing days at both T1 and T2 to obtain an average measurement of emotional support across all testing days per timepoint. For high school students, the Cronbach’s α_day 1_ was 0.74 and 0.78 for friends, and 0.74 and 0.83 for family members at T1 and T2, respectively. For university students, the Cronbach’s α_day 1_ was 0.59 and 0.63 for friends, and 0.71 and 0.78 for family members at T1 and T2, respectively.

#### Experimental giving behavior

Pandemic Dictator Games (DG) were used to measure familiarity, need, and deservedness effects on giving to COVID-19 related targets^6,20^. In five one-shot Dictator Games in randomized order, participants divided 10 coins between themselves and 5 different individuals (i.e., targets). These targets included an unknown peer, a friend (familiar target), a medical doctor working at a hospital (deserving target), a COVID-19 patient, and an individual with a poor immune system (targets in need). The exact identity of these targets remained unknown to the participants. Participants were explained that the coins were as valuable for themselves as for other individuals and that the divisions of the coins were hypothetical but should be treated as if they were real outcomes for self and other (i.e., there was no actual payment). The Dictator Games were administered four times: on the first and last day of the testing period at T1, on the first day at T2, and at the follow-up session T3. Giving behavior was coded as the absolute number of donated coins (range 0–10) for each target, for all four timepoints.

### Emotional and cognitive factors

#### Social reward sensitivity

Individual differences in the value of social rewards were assessed with the Prosocial Interactions subscale (5 items; example item ‘I enjoy making someone feel happy’) of the Social Reward Questionnaire for the university students (SRQ) and the Social Reward Questionnaire—Adolescent version for high school students (SRQ-A)^30,31^. Using a 7-point Likert scale ranging from 1 (strongly disagree) to 7 (strongly agree), participants were asked to rate several statements on interactions with other people (e.g., friends, family members, and acquaintances). A mean score of the five items was computed. The SRQ and SRQ-A were only administered at T1. Cronbach’s α was 0.82 for high school students and 0.79 for university students.

#### Executive functioning

Executive functioning was measured using the Web-Based Executive Function Questionnaire (Webexec)^32^, which consists of six items. An example item is ‘Do you find it difficult to keep your attention on a particular task?’. Participants rated on a 4-point scale (no problems experienced—a great many problems experienced) whether the items generally applied to them. At both timepoints, a mean score of the six items was computed. For high school students, the Cronbach’s α was 0.83 and 0.86 at T1 and T2, respectively. For university students, the Cronbach’s α was 0.84 and 0.85 at T1 and T2, respectively.

#### General willingness to contribute to society

The general willingness to contribute to society was assessed with the General Contribution to Society questionnaire (GCS)^6^. Using a scale from 1 (not at all) to 10 (very much), participants were asked to rate whether two statements applied to them, namely ‘I think it is important to contribute to society’ and ‘I think it is important to make an effort for the people around me’. We explained that contributions to society can take many different forms, such as (volunteer) work. For both timepoints, a mean score of the two statements was computed. For high school students, Cronbach’s α was 0.72 and 0.70 at respectively T1 and T2. For university students, Cronbach’s α was 0.72 and 0.69 at respectively T1 and T2.

#### Altruism

Altruism, defined as helping others without potentially receiving a direct and/or explicit reward, was measured with **t**he Altruism subscale (6 items) of Prosocial Tendencies Measure—Revised (PTM-R)^33^. An example item is ‘I often help even if I don’t think I will get anything out of helping’. On the fifth day of the testing period, participants were asked to rate the extent to which the items applied to them on a scale from 1 (does not describe me at all) to 5 (describes me very well). A mean score of the six items was computed. PTM-R was only administered at T1. Cronbach’s α was 0.65 for high school students and 0.68 for university students.

Two additional individual difference measures (i.e., self-control and emotional awareness) are described in Supplementary S2.

## Results

Descriptive statistics can be found in Table 1. Correlation matrices are shown in Tables 2, 3 and 4. The reliability of measures across timepoints can be found in Table 5. Because the Mauchly’s test of sphericity indicated violations of sphericity (*p* < 0.001), we reported all effects using the Greenhouse–Geisser correction. While the preregistration mentions separate analyses for the two samples (i.e., high school and university students), we deviated from this preregistered analysis plan to keep the present study as concise and clear as possible (see https://osf.io/h5x2a/).

**Table 1:** Descriptive statistics of giving, daily emotional support, altruism, self-control, emotion awareness, general contributions to society, executive functioning, and social reward sensitivity at all timepoints (T1, T2, and T3).

Measure		High School Students						University Students					
	# items	*N*	Min score	Max score	Mean	95% CI Lower Bound	95% CI upper bound	*N*	Min score	Max score	Mean	95% CI lower bound	95% CI upper bound
**Dictator game T1 day 1**													
Unknown peer	1	468	0.00	8.00	3.35	3.19	3.52	389	0.00	10.00	3.27	3.06	3.47
Friend	1	468	0.00	10.00	5.06	4.96	5.16	389	0.00	10.00	5.08	4.95	5.21
Doctor at a hospital	1	467	0.00	10.00	6.86	6.65	7.07	389	0.00	10.00	6.76	6.53	7.00
COVID-19 patient	1	467	0.00	10.00	6.54	6.30	6.78	389	0.00	10.00	6.20	5.95	6.46
Individual with poor immune system	1	467	0.00	10.00	6.04	5.82	6.27	389	0.00	10.00	6.02	5.79	6.25
**Dictator game T1 day 10**													
Unknown peer	1	417	0.00	10.00	3.34	3.16	3.52	335	0.00	10.00	3.30	3.07	3.53
Friend	1	417	0.00	10.00	5.03	4.92	5.15	335	0.00	10.00	5.00	4.88	5.12
Doctor at a hospital	1	417	0.00	10.00	6.68	6.46	6.90	335	0.00	10.00	6.51	6.26	6.76
COVID-19 patient	1	417	0.00	10.00	6.02	5.77	6.27	335	0.00	10.00	6.16	5.90	6.42
Individual with poor immune system	1	417	0.00	10.00	5.51	5.27	5.74	335	0.00	10.00	5.92	5.69	6.16
**Dictator game T2 day 1**													
Unknown peer	1	238	0.00	9.00	3.43	3.19	3.66	235	0.00	8.00	3.19	2.93	3.44
Friend	1	238	0.00	10.00	5.13	4.95	5.31	235	0.00	10.00	4.96	4.81	5.12
Doctor at a hospital	1	238	0.00	10.00	6.54	6.25	6.83	235	0.00	10.00	6.23	5.91	6.54
COVID-19 patient	1	238	0.00	10.00	5.80	5.50	6.10	235	0.00	10.00	5.37	5.06	5.68
Individual with poor immune system	1	238	0.00	10.00	5.61	5.32	5.90	235	0.00	10.00	5.85	5.56	6.14
**Dictator game T3**													
Unknown peer	1	205	0.00	7.00	3.52	3.27	3.78	168	0.00	8.00	3.02	2.71	3.33
Friend	1	205	0.00	10.00	5.15	4.96	5.34	168	0.00	9.00	4.92	4.75	5.08
Doctor at a hospital	1	205	0.00	10.00	6.60	6.29	6.90	168	0.00	10.00	5.82	5.44	6.20
COVID-19 patient	1	205	0.00	10.00	5.16	4.83	5.49	168	0.00	10.00	5.08	4.70	5.45
Individual with poor immune system	1	205	0.00	10.00	5.44	5.15	5.72	168	0.00	10.00	5.58	5.21	5.96
**Daily emotional support T1**													
Friends	3	481	0.00	5.00	2.29	2.19	2.38	397	0.00	5.00	2.30	2.20	2.40
Family	3	481	0.00	5.00	1.66	1.57	1.76	397	0.00	5.00	1.90	1.80	2.01
**Daily emotional support T2**													
Friends	3	250	0.00	5.00	3.01	2.87	3.15	240	0.23	5.00	2.73	2.60	2.85
Family	3	242	0.00	5.00	2.21	2.06	2.37	240	.037	5.00	2.41	2.28	2.54
**Emotional and cognitive moderators**													
Altruism	6	405	1.83	5.00	3.80	3.73	3.86	322	2.17	5.00	4.04	3.97	4.10
General contributions to society T1	2	411	1.00	10.00	6.88	6.71	7.06	324	2.50	10.00	7.23	7.05	7.42
General contributions to society T2	2	211	1.00	10.00	7.27	7.05	7.48	197	2.00	10.00	7.29	7.08	7.50
Executive functioning T1	6	410	1.00	4.00	2.03	1.96	2.09	324	1.00	4.00	2.11	2.03	2.18
Executive functioning T2	6	211	1.00	4.00	2.07	1.98	2.16	197	1.00	4.00	2.09	1.99	2.19
Social reward sensitivity	5	407	1.00	7.00	6.15	6.07	6.23	323	1.00	7.00	6.39	6.32	6.47

The minimum and maximum scores indicate the actual responses (i.e., not potential responses) to the measures.

**Table 2:** Bivariate correlations among all variables used in the statistical analyses (at T1).

	Dictator game day 1					Dictator game day 10				
	Unknown peer	Friend	Doctor at a hospital	COVID-19 patient	Individual with poor immune system	Unknown peer	Friend	Doctor at a hospital	COVID-19 patient	Individual with poor immune system
Age (*N* = 888)	− 0.013	− 0.002	− 0.049	− 0.093**	− 0.056	− 0.022	− 0.019	− 0.079*	− 0.011	00.17
Gender (*N* = 864)	0.132**	0.114**	0.197**	0.156**	0.179**	0.109**	0.067	0.157**	0.124**	0.166**
SES (*N* = 823)	0.003	− 0.047	− 0.046	− 0.029	0.005	0.028	− 0.039	− 0.081*	− 0.047	− 0.015
**Emotional and cognitive moderators**										
Altruism	0.203**	0.225**	0.144**	0.185**	0.206**	0.164**	0.152**	0.110**	0.181**	0.191**
Executive functioning	0.054	0.048	0.072	0.016	0.037	0.057	0.057	0.079*	0.020	0.029
General contributions to society	0.161**	0.180**	0.189**	0.211**	0.224**	0.164**	0.122**	0.203**	0.188*	0.279**
Social reward sensitivity	0.102**	0.122**	0.126**	0.171**	0.099**	0.129**	0.086*	0.123**	0.094*	0.112**
**Emotional support (*N* = 878)**										
Friends	0.105**	0.197**	0.187**	0.180**	0.166**	0.109**	0.157**	0.154**	0.155**	10.70**
Family	0.018	0.064	0.122**	0.139**	0.167**	0.015	− 0.001	0.073*	0.137**	0.194**
**Dictator game day 1 (*N* = 857)**										
Unknown peer	–									
Friend	0.426**	–								
Doctor at a hospital	0.362**	0.399**	–							
COVID-19 patient	0.395**	0.410**	0.635**	–						
Individual with poor immune system	0.403**	0.402**	0.621**	0.705**	–					
**Dictator game day 10 (*N* = 752)**										
Unknown peer	0.703**	0.385**	0.283**	0.343**	0.358**	–				
Friend	0.353**	0.694**	0.318**	0.344**	0.335**	0.448**	–			
Doctor at a hospital	0.282**	0.313**	0.717**	0.532**	0.522**	0.347**	0.329**	–		
COVID-19 patient	0.374**	0.316**	0.544**	0.748**	0.637**	0.415**	0.316**	0.617**	–	
Individual with poor immune system	0.387**	0.329**	0.481**	0.582**	0.702**	0.488**	0.374**	0.587**	0.715**	–

*p < 0.05, **p < 0.01.

**Table 3:** Bivariate correlations among all variables used in the statistical analyses (at T2).

	Dictator game				
	Unknown peer	Friend	Doctor at a hospital	COVID-19 patient	Individual with poor immune system
Age (*N* = 473)	− 0.065	− 0.096*	− 0.115*	− 0.124**	− 0.034
Gender (*N* = 472)	− 0.006	0.053	0.148**	0.105*	0.139**
SES (*N* = 446)	0.084	0.021	0.012	− 0.008	0.053
**Emotional and cognitive moderators**					
Executive functioning	0.067	0.018	0.076	− 0.007	0.056
General contributions to society	0.154**	0.225**	0.141**	0.226**	0.205**
**Emotional support (*N* = 470)**					
Friends	0.158**	0.208**	0.194**	0.194**	0.180**
Family	0.105*	0.097*	0.146**	0.188**	0.149**
**Dictator game (*N* = 473)**					
Unknown peer	–				
Friend	0.406**	–			
Doctor at a hospital	0.374**	0.414**	–		
COVID-19 patient	0.492**	0.460**	0.621**	–	
Individual with poor immune system	0.506**	0.464**	0.658**	0.729**	–

*p < 0.05, **p < 0.01.

**Table 4:** Bivariate correlations among all variables used in the statistical analyses (at T3).

	Dictator game				
	Unknown peer	Friend	Doctor at a hospital	COVID-19 patient	Individual with poor immune system
Age (*N* = 370)	− 0.108*	− 0.100	− 0.161**	− 0.040	0.011
Gender (*N* = 370)	− 0.002	0.041	0.208**	0.096	0.124*
SES (*N* = 355)	0.020	− 0.046	− 0.062	− 0.011	− 0.005
**Dictator game (*N* = 373)**					
Unknown peer	–				
Friend	0.439**	–			
Doctor at a hospital	0.334**	0.294**	–		
COVID-19 patient	0.550**	0.393**	0.506**	–	
Individual with poor immune system	0.601**	0.396**	0.543**	0.693**	–

*p < 0.05, **p < 0.01.

**Table 5:** Intraclass correlation coefficients (ICC) across all timepoints (T1, T2, and T3).

	ICC	95% CI lower bound	95% CI upper bound
**Dictator game (T1 day 1, T1 day 10, T2, T3)**			
Unknown peer	0.88	0.86	0.90
Friend	0.84	0.81	0.87
Doctor at a hospital	0.84	0.81	0.87
COVID-19 patient	0.82	0.79	0.85
Individual with poor immune system	0.86	0.83	0.88
Daily emotional support (T1, T2)*			
Friends	0.95	0.93	0.96
Family	0.95	0.94	0.96
**Emotional and cognitive moderators**			
General contributions to society (T1, T2)	0.71	0.65	0.76
Executive functioning (T1, T2)	0.80	0.76	0.84

*ICC of daily emotional support based on 10 daily measures at each timepoint (T1 and T2).

### Daily emotional support toward friends and family

#### Differences in daily emotional support at T1

**Figure 3:**
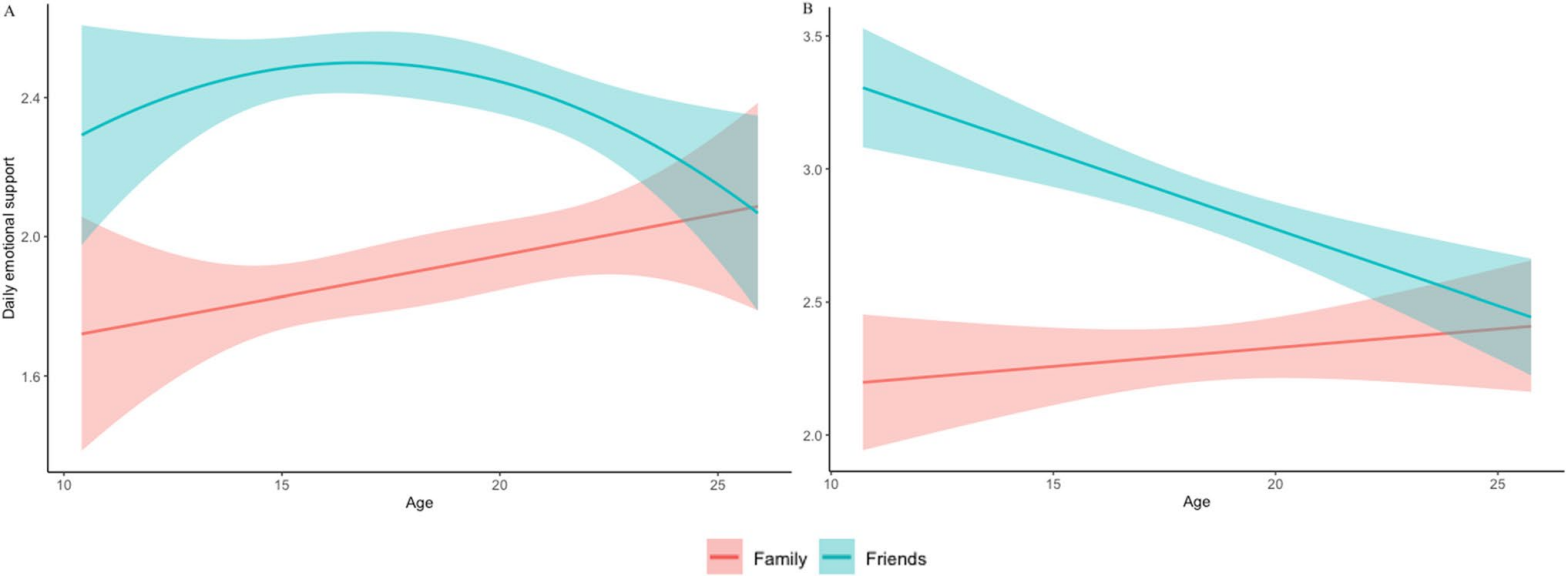
Mean levels of daily emotional support toward friends and family members in May 2020 at T1 (**A**) and in November 2020 at T2 (**B**) during the COVID-19 pandemic. At T1, adolescents displayed higher levels of emotional support to friends compared to family, and this difference was especially pronounced in late adolescence. At T2, adolescents again showed higher levels of emotional support to friends compared to family, with a linear decrease in emotional support to friends with age. Band widths indicate 95% confidence intervals. Note that the y-axis is scaled per time point.

To examine differences in daily emotional support toward friends and family members at the first timepoint (T1), we performed a repeated measures ANOVA with target (friends, family) as a within-subject factor. The analysis was based on *N* = 878 and showed a main effect of target, *F*(1, 805) = 93.79, *p* < 0.001, ${\eta }_{p}^{2}$ = 0.10, indicating more emotional support towards friends (*M* = 2.21, 95% CI [2.08, 2.33]) than family (*M* = 1.66, 95% CI [1.53, 1.79]), The analysis further yielded an interaction effect between target and quadratic age, *F*(1, 805) = 7.14, *p* = 0.008, ${\eta }_{p}^{2}$ = 0.01. As can be seen in Fig. 3, the relation between age and emotional support to friends was non-linear, with a peak during late adolescence, *F*(1, 812) = 4.10, *p* = 0.043, ${\eta }_{p}^{2}$ = 0.01. Emotional support to family increased linearly with age, *F*(1, 812) = 5.14, *p* = 0.024, ${\eta }_{p}^{2}$ = 0.01. There was also a main effect of gender, *F*(1, 805) = 4.52, *p* = 0.034, ${\eta }_{p}^{2}$ = 0.01, indicating that females on average (*M* = 2.06, 95% CI [1.94, 2.18]) showed more daily emotional support than males (*M* = 1.81, 95% CI [1.61, 2.01]). There were no gender by target interactions.

Because the target by age interaction effects may be explained by adolescents’ living situation (e.g., living with friends or family members), we also tested exploratory the effects of roommates on daily emotional support. We performed a similar repeated measures ANOVA with target (friends, family) as a within-subjects factor. Living situation at T1 (alone, family, peers, romantic partner) was added as between-subjects factors with age and gender as covariates, while controlling for SES. Separate analyses with linear and quadratic age as covariates (both *N* = 801) showed no main effect of living situation and no interaction with target (all *p*’s > 0.05). However, the interaction effect between target and quadratic age was no longer significant after adding living situation as control variable (*p* > 0.05). We did find a significant interaction effect between target and linear age, such that emotional support to family increased linearly with age, *F*(1, 811) = 41.64, *p* = 0.020, ${\eta }_{p}^{2}$ = 0.01.

We then examined whether the same patterns were observed at the second timepoint (T2; *N* = 494). We replicated the general findings of T1, although at T1 emotional support showed a quadratic peak whereas at T2 this effect was linear, such that emotional support to friends was higher among young adolescents (see Fig. 3; Supplementary S4).

#### Longitudinal differences in daily emotional support

**Figure 4:**
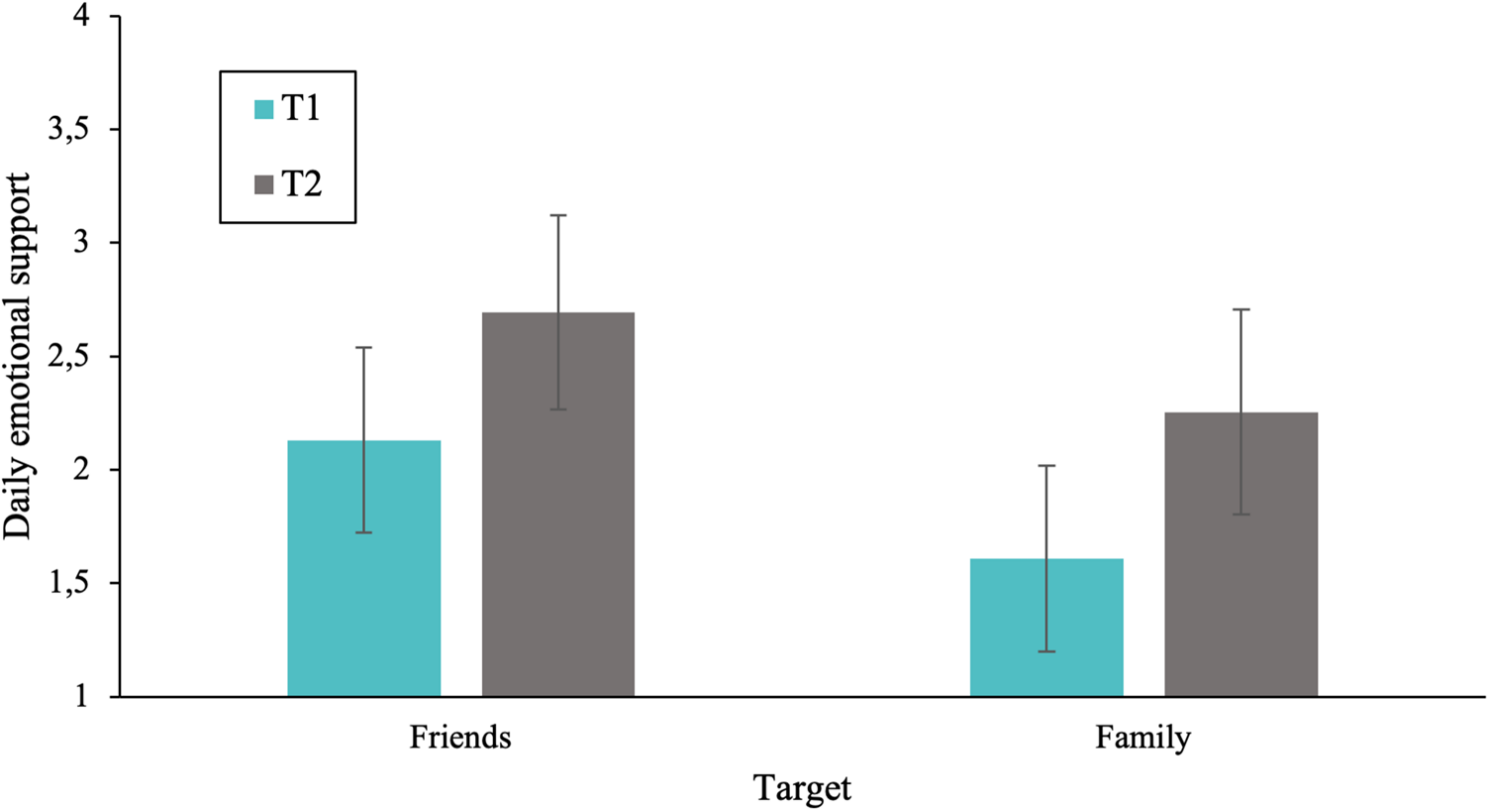
Mean levels of daily emotional support toward friends and family members at two timepoints (T1: May 2020 and T2: November 2020) during the COVID-19 pandemic. Adolescents showed an increase in daily emotional support from T1 to T2. Error bars denote 95% confidence intervals.

To examine the longitudinal trajectory of daily emotional support toward friends and family members, we performed repeated measures ANOVA with time (T1, T2) and target (friends, family) as within-subject factors, while controlling for SES, including only the participants who participated at both timepoints. In this analysis, we observed a main effect of time, *F*(1, 442) = 32.72, *p* < 0.001, ${\eta }_{p}^{2}$ = 0.07. As shown in Fig. 4, results indicated higher levels of emotional support towards friends and family at T2 (*M* = 1.87, 95% CI [1.68, 2.01]) compared to T1 (*M* = 2.47, 95% CI [2.28, 2.67]). The interaction between time and target was not significant.

### Giving toward unfamiliar peers, friends, and COVID-19 targets

#### Differences in giving behavior at T1

To examine giving behavior toward different targets at the first timepoint (T1), we performed a repeated measures ANOVA with DG target (unknown peer, friend, medical doctor, COVID-19 patient, and individual with a poor immune system) and DG time (day 1, day 10) as within-subject factors. Age and gender were added as covariate and between-subject factor of interest. SES was added as a control covariate.

**Figure 5:**
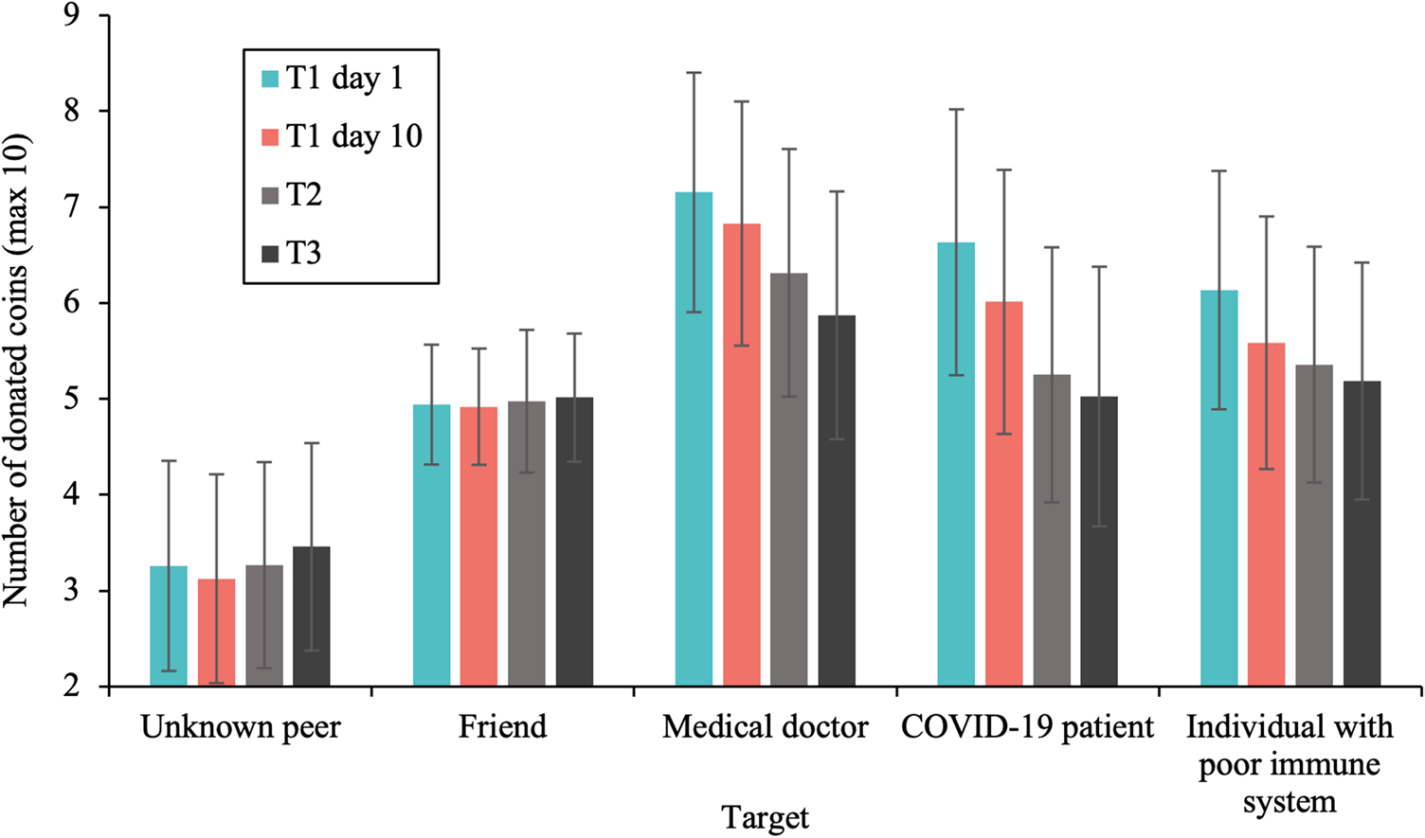
Giving behavior toward all targets in the Dictator Game for all timepoints (T1 day 1, T1 day 10, T2, and T3). Most coins were donated to COVID-19 related targets (i.e., medical doctor, COVID-19 patient, and individual with poor immune system), followed by a friend and an unknown peer. Adolescents showed a decrease in giving behavior to the COVID-19 related targets across time. Error bars denote 95% confidence intervals.

The analysis (*N* = 738) resulted in a main effect of target, *F*(3.29, 2273.17) = 34.26, *p* < 0.001, ${\eta }_{p}^{2}$ = 0.05. Post-hoc pairwise Bonferroni corrected comparisons revealed that all targets were significantly different from each other in terms of given coins (*p*’s < 0.001; see also Fig. 5). Most coins were given to a doctor at a hospital (*M* = 6.95, 95% CI [6.70, 7.19]), followed by a COVID-19 patient (*M* = 6.30, 95% CI [6.03, 6.57]), followed by an individual with a poor immune system (*M* = 5.86, 95% CI [5.61, 6.11]); i.e., the targets that were deserving and in need). Fewer coins were donated to a friend (familiar target; *M* = 5.05, 95% CI [4.93, 5.18]) and the least number of coins to an unknown peer (*M* = 3.26, 95% CI [3.05, 3.46]). The RM ANOVA also resulted in a main effect of gender, *F*(1, 691) = 7.37, *p* < 0.001, ${\eta }_{p}^{2}$ = 0.01, showing that females donated more coins than males, and an interaction between target and gender, *F*(3.29, 2273.17) = 3.72, *p* = 0.009, ${\eta }_{p}^{2}$ = 0.01. Females showed higher giving behavior toward all individual targets compared to males (all *p*s < 0.05), but this pattern was most pronounced in giving behavior towards the COVID-19 targets.

The RM ANOVA further resulted in a main effect of DG time, *F*(1, 691) = 11.38, *p* = 0.001, ${\eta }_{p}^{2}$ = 0.02, and an interaction effect between DG time and target, *F*(3.74, 2587.07) = 7.61, *p* < 0.001, ${\eta }_{p}^{2}$ = 0.01. The analysis further yielded main effects of linear age, *F*(1, 691) = 5.00, *p* = 0.026, ${\eta }_{p}^{2}$ = 0.01, an interaction effect between time and linear age, *F*(1, 691) = 5.36, *p* = 0.021, ${\eta }_{p}^{2}$ = 0.01, and a three-way interaction effect between time, target, and linear age, *F*(3.74, 2587.07) = 5.93, *p* < 0.001, ${\eta }_{p}^{2}$ = 0.01.

**Figure 6:**
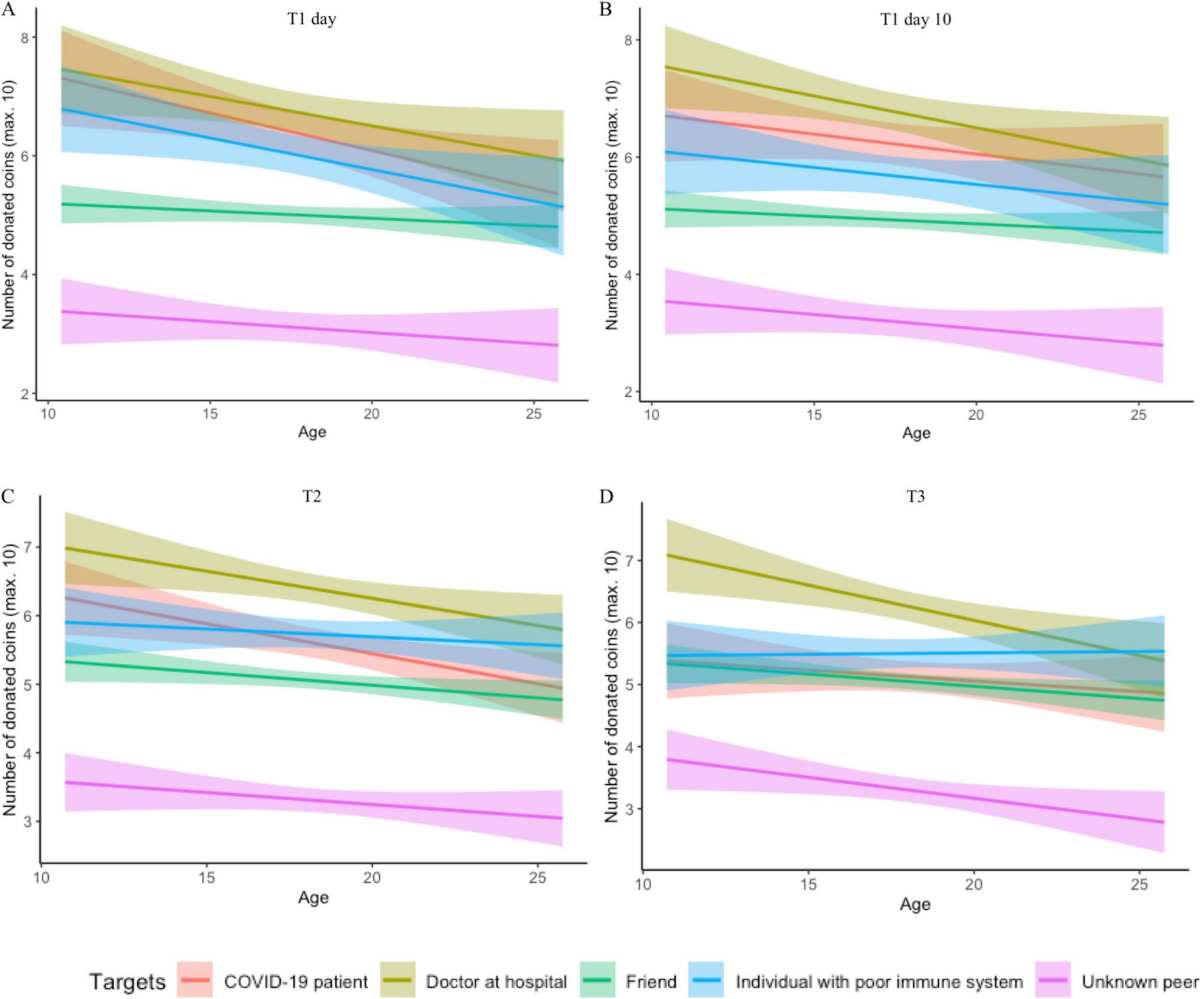
Age effects on giving behavior, as indicated by the mean levels of donated coins toward all targets in the Dictator Game for T1 day 1 ((**A**); *N* = 857), T1 day 10 ((**B**); *N* = 752), T2 ((**C**); *N* = 473), and T3 ((**D**); *N* = 373). With age, adolescents showed a decrease in giving behavior to COVID-19 targets. Giving behavior towards friends and unknown peers remained stable with age across time. Band widths indicate 95% confidence intervals.

This 3-way interaction was unpacked in three steps. First, at both timepoints giving behavior towards medical doctors decreased with age (*B*_day 1_ =  − 0.06, *p*_day 1_ = 0.021; *B*_day 10_ =  − 0.07, *p*_day 10_ = 0.004), with no effects of time. Second, there were no age-related differences across time in giving towards friends and unknown peers (all *p*s > 0.05) nor any interactions with time. Third, the interaction effect with time and age was driven by giving behavior towards two targets: At the first timepoint, giving behavior toward COVID-19 patients (*B* =  − 0.10, *p* = 0.001) and individuals with a poor immune system (*B* =  − 0.06, *p* = 0.033) decreased with age. However, at the second timepoint, there was no longer an age-related decrease in giving to COVID-19 patients and individuals with a poor immune system (see Fig. 6).

The main effect of target, the main and interaction effects of age, as well as the interaction between target and gender, were replicated at T2 (see Supplementary S4).

#### Longitudinal differences in giving behavior

To examine the longitudinal trajectory of giving behavior toward different targets, we performed a repeated measures ANOVA with DG time including all timepoints (T1 day 1, T1 day 10, T2, T3) and DG target (unknown peer, friend, medical doctor, COVID-19 patient, and individual with a poor immune system) as within-subjects factors. Age and gender were added as covariate and between-subject factor, while SES was added as a control covariate. This analysis was based on *N* = 373 participants. Whereas the main effect of time was non-significant (*p* > 0.05), the ANOVA yielded an interaction effect between target and time, *F*(9.56, 2742.18) = 1.87, *p* = 0.048, ${\eta }_{p}^{2}$ = 0.01. Post hoc comparisons revealed that giving behavior to COVID-19 related targets decreased with time (all *p*s < 0.01; see also Fig. 5). Time effects were not significant for friends (*p* = 0.892) and unknown peers (*p* = 0.273).

### Associations between daily emotional support and giving

#### Effect of daily emotional support on giving behavior at T1

**Figure 7:**
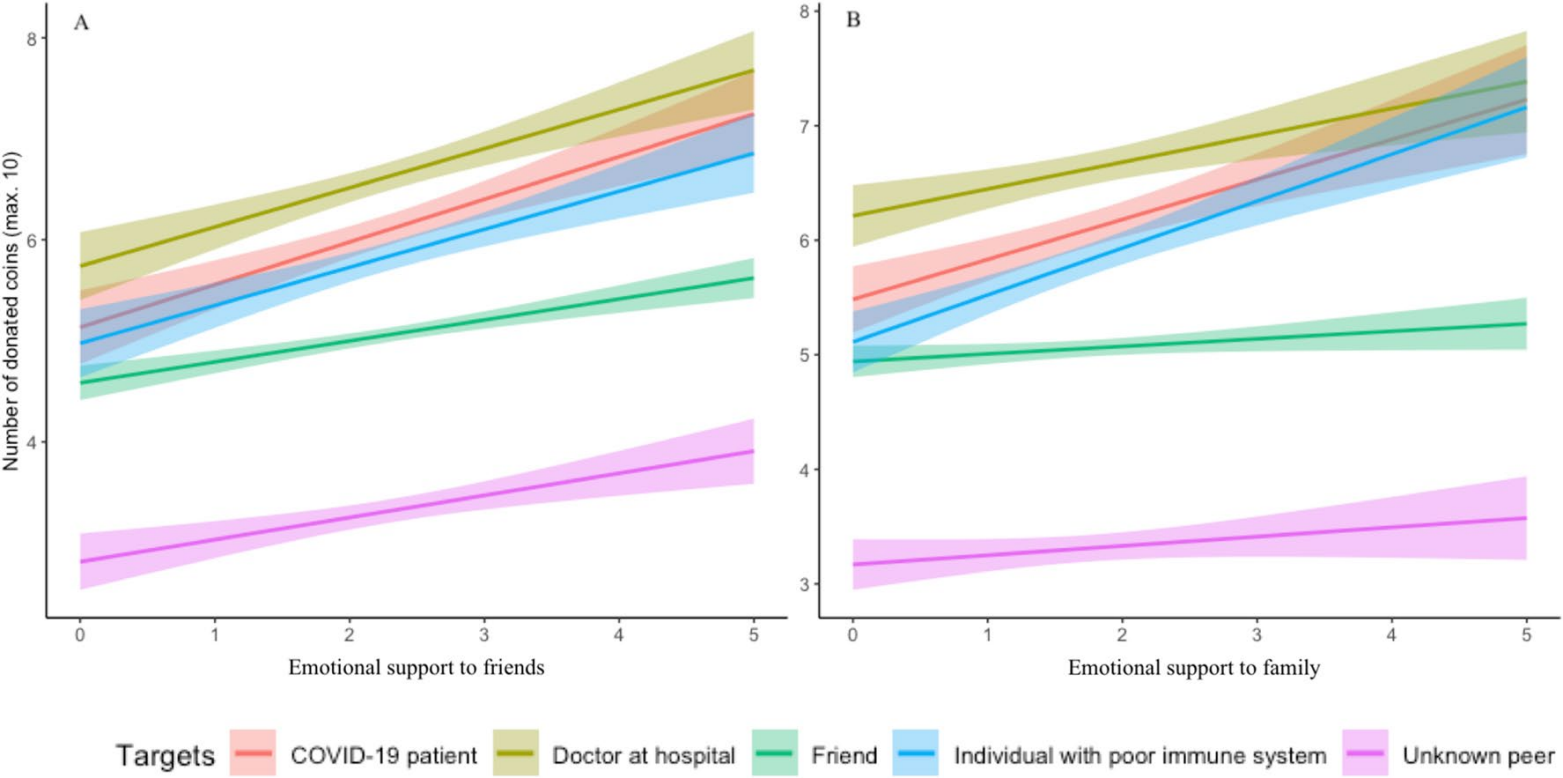
Interaction effect between emotional support to friends (**A**) and family (**B)** as measured at T1 and giving toward all targets, averaged across the first two timepoints (T1 day 1 with *N* = 857, T1 day 10 with *N* = 752, and T2 with *N* = 473). Higher levels of daily emotional support towards friends were associated with more donations to others. Adolescents with higher levels of emotional support towards family donated more coins to COVID-19 patients and individuals with a poor immune system, but not to the other targets. Band widths indicate 95% confidence intervals.

To examine the effect of daily emotional support on giving behavior at the first timepoint (T1), a repeated measures ANOVA was conducted with DG time and DG target as within-subject factors and with emotional support to each target (friends, family) as covariate, controlling for gender, age and SES. The analysis (*N* = 698) showed a main effect of emotional support to friends on donations, *F*(1, 691) = 8.81, *p* = 0.003, ${\eta }_{p}^{2}$ = 0.02, such that adolescents with higher levels of daily emotional support towards friends donated more coins to others (see Fig. 7). There were no interaction effects with target or time.

The analysis yielded no main effect of emotional support to family (*p* = 0.546), but there was an interaction effect between target and emotional support to family, *F*(3.32, 2294.38) = 10.96, *p* < 0.001, ${\eta }_{p}^{2}$ = 0.02. Emotional support to family was significantly associated with giving to an individual with a poor immune system (*B* = 0.37, *p* < 0.001), but not significantly associated with giving to an unknown peer (*B* =  − 0.14, *p* = 0.072), a friend (*B* =  − 0.09, *p* = 0.059), a doctor at a hospital (*B* = 0.11, *p* = 0.221), or a COVID-19 patient (*B* = 0.18, *p* = 0.071). As shown in Fig. 7, these results show that those who displayed more emotional support towards family donated more coins to individuals with a poor immune system (i.e., to those in need), while no such effects were found for the other targets. There were no interaction effects with time.

As discussed in Supplementary S4, the main effect of emotional support to friends and the interaction effect between target and emotional support to family were replicated at T2. Given significant correlations between the two covariates (i.e., emotional support to the two targets), we performed similar analyses using an average score of the two targets (see Supplementary S5).

### Emotional and cognitive variables—moderators

#### Moderating effects of emotional and cognitive variables at T1

Finally, we examined moderating effects of emotional and cognitive variables on the association between daily emotional support and giving behavior. For each moderator, we performed a separate repeated measures ANOVA with DG time and DG target as within-subject factors, and emotional support to friends or family and the moderator as predictors (allowing for interactions between emotional support and the moderator). We controlled for gender, SES and age by adding these as between-subjects factors and covariates, respectively.

#### Social reward sensitivity

Regarding SRQ, we observed a three-way interaction effect between time, target and SRQ on giving behavior, *F*(3.74, 2323.65) = 6.41, *p* < 0.001, ${\eta }_{p}^{2}$ = 0.01. To follow-up on this effect, we performed separate repeated measures ANOVAs for both timepoints. This analysis showed an interaction between target and sensitivity to prosocial rewards on giving on day 1, but not day 10. A follow-up analysis on the interaction between target and sensitivity to prosocial rewards on giving on day 1 showed that higher levels of social reward sensitivity were associated with higher giving to a COVID-19 patient (*B* = 0.75, *p* = 0.004), but not towards other targets (*ps* > 0.05).

We also observed a four-way interaction between time, target, SRQ, and emotional support to friends, *F*(3.74, 2457.26) = 4.07, *p* = 0.003, ${\eta }_{p}^{2}$ = 0.01. Following up this effect by performing a repeated measures ANOVA with emotional support towards friends as single predictor in interaction with SRQ as moderator, we found an interaction between target, emotional support towards friends, and social reward sensitivity on giving to medical doctors on day 1 (*B* = 0.19, *p* = 0.045), but not to other targets (all *p*s > 0.05). These results suggest that the positive relation between emotional support towards friends and giving to medical doctors is stronger for those scoring higher on social reward sensitivity on day 1.

#### Executive functions

No (moderating) effects of executive functioning were detected.

#### General willingness to contribute to society

We found an interaction effect between GCS and emotional support towards friends, *F*(1, 622) = 3.95, *p* = 0.047, ${\eta }_{p}^{2}$ = 0.01, such that adolescents who are generally more willing to contribute to society show a stronger positive relation between emotional support to friends and giving to others. No other interaction effects with GCS were detected.

#### Altruism

No main or moderating effects of altruism were found.

See Supplementary S4 for the replication at T2 of the moderating effect of societal awareness on the relation between emotional support and giving.

## Discussion

The aim of the current study was to examine the effects of the COVID-19 pandemic on the extent to which adolescents provided emotional support towards friends and family and the relation to giving to unknown others, friends or deserving pandemic-related targets. As expected, we demonstrated that providing daily emotional support toward friends was highest in the late teen-age years, whereas providing emotional support to families gradually increased between ages 10 and 25 years. Moreover, emotional support to both friends and family members increased between May 2020 and November 2020. Second, we observed higher experimental giving to pandemic targets (medical doctors, COVID-19 patients, and individuals with a poor immune system), intermediate giving to friends and lowest giving to unknown others, reinforcing that adolescents were aware of the needs of others during the pandemic^6^. Moreover, adolescents’ daily emotional support actions to friends and family during the COVID-19 pandemic were associated with more experimental giving to medical doctors, COVID-19 patients, and individuals with a poor immune system. Emotional support to family was most strongly related to experimental giving to individuals with a poor immune system. These findings support the hypothesis that social experiences are of significant value in adolescence and impact social behavior towards deserving targets in the pandemic.

We observed that emotional support towards friends is highest in mid-to-late adolescence relative to early adolescence and young adulthood, which is consistent with pre-pandemic behavior^13^. These findings show that also in times of social distancing adolescents seek out opportunities to provide support to friends. In contrast, emotional support towards family showed a slowly emerging pattern across adolescence, resulting in similar levels of emotional support to friends and family by early adulthood. At the second timepoint, there was no longer a developmental difference in emotional support to family, but emotional support to friends remained higher than to family, especially in early adolescence. One possibility is that the divergence in emotional support to friends and family in early to mid-adolescence is associated with the need to gain independence, whereas reciprocal relationships with family are valued more in young adulthood^15,25^.

A unique aspect of this study was that we followed the same individuals during the pandemic. Time-related analysis demonstrated that despite social distancing, emotional support to friends and family increased during the pandemic, whereas giving behavior to friends was relatively stable. Possibly, emotional support to friends was associated in higher social reward value associated with gaining positive attention from others for adolescents relative to children and adults^14,15^. The increase in providing emotional support to friends and families was opposite from our hypotheses, where we predicted a decrease due to social distancing and restrictions to spend time together. Possibly, emotional support was expressed through calling, social media or texting, or through other types of helping and support. Future studies should further reveal how specific types of helping was expressed during the pandemic crisis.

Our next aim was to examine how daily emotional support was associated with experimental giving behavior to peers, friends, and COVID-19 targets. There was a general decreasing age pattern in giving to COVID-19 targets, possibly reflecting stronger norm-based giving in young adolescents which decreased during adolescence^34^, and which decreased over the course of the pandemic. Norm-based giving is defined as an equity preference, which is relatively stronger in childhood and decreases during adolescence, possibly because of an increase in understanding of the needs or investments of others^34^. Indeed, prior studies reported a shift during adolescence from norm-based giving to giving that reflects strategic perspectives for self and others in early adulthood^35,36^. An interesting finding in previous research is that over the course of adolescence, there is a larger differentiation in giving to friends versus strangers, specifically showing more giving to friends and less to disliked or unknown others^20,27^. Whereas prior studies demonstrated an age-related increase in target differentiation^20^, the current study showed that target differentiation can also decrease such that deserving or vulnerable targets are treated more similar to friends with increasing age^27,37^. Future research could look further into these age effects on target differentiation in combination with pre-pandemic data or by testing giving to various targets when the pandemic comes to an end. Future studies should also include giving to family members as an addition condition, to examine the relation between daily support to family and giving to family^18^.

An important aim of this study was to examine how daily prosocial actions were related to giving, and whether these relations were moderated by individual differences, such as social reward sensitivity. Daily emotional support to friends predicted experimental giving to others, possibly reflecting that support directed to friends fosters perspective taking and kindness^2,15^. Emotional support to family, in contrast, predicted more experimental giving only to individuals with a poor immune system, possibly showing that family relations influence giving towards vulnerable others^25,38^. The relations with social reward sensitivity were complex, but an interesting relation was found between social reward sensitivity and giving to COVID-19 patients and medical doctors, partly validating the experimental giving paradigm. Given that these findings suggest that declining prosocial actions may impact the developmental need to contribute to needs of others^2,3^, it is crucial that adolescents are provided with opportunities for prosocial actions, not only during the pandemic but also at post-pandemic times. Creating these opportunities in the adolescents’ social environment, such as in the school setting and in family and peer relationships, helps adolescents to provide support to others, which, in turn, is beneficial for their wellbeing^24^.

This study had several strengths, including a large sample size and data collection during multiple timepoints during the pandemic. The use of daily diaries provided a richer assessment of daily experiences and we could replicate relations across two timepoints. However, there were also limitations, including relatively high attrition across timepoints. Second, the young adults in this study were relatively highly educated. Third, the Dictator Game was administered as a hypothetical dilemma game, therefore it may limit the possibility to generalize the findings to real life giving. Even though prior studies supported the external validity of Dictator Games by showing similar results using economic games as well as self-reported prosocial behaviors^39^, a meta-analysis previously showed that in general individuals are more generous in hypothetical than real-life situations^40^. Finally, given the longitudinal design of the current study, changes in governmental restrictions occurred during the study (e.g., campus closed for university students but schools open for high school students). These changes may have affected adolescents’ opportunities for providing support to friends and family differently at the multiple timepoints of the study. It was a specific aim of this study to examine giving under pandemic restrictions, but it should be noted that there may be confounds related to the opportunities that individuals had to give support to others.

Taken together, giving to friends and providing emotional support followed different developmental trajectories. Whereas daily emotional support peaked in mid-adolescence, experimental giving to friends was relatively stable across age. Moreover, daily emotional support increased over the course of the pandemic. One explanation is that giving to friends is driven by other factors in addition to providing support, such as reciprocity and egalitarian relationships^34^. Providing daily emotional support to friends may be associated with vicarious reward feelings or feeling an intrinsic value of being able to contribute to the needs of others^19,24^. This is consistent with the notion that prosocial behaviors have varying antecedents, consequences, and developmental trajectories, and that prosociality should therefore be understood as a multi-dimensional construct^15^. Intriguingly, daily prosocial actions were related to general contributions to society in adolescence. Together, the results of this study demonstrate the importance of providing prosocial opportunities which may reinforce the development of kind and supporting relationships not only with friends and family, but also with those who are vulnerable in society.

## Supplementary Information

Supplementary Information.

## Data Availability

The study was preregistered and materials for this study have been made publicly available on Open Science Framework (OSF; see https://osf.io/h5x2a/). Data are made publicly at the EUR data repository (see 10.25397/eur.c.5809043).
