# Supplementary material for: Daily prosocial actions during the COVID-19 pandemic contribute to giving behavior in adolescence
Source: Sci Rep. 2022 May 6;12:7458. doi: 10.1038/s41598-022-11421-3 (PMC9075144; doi:10.1038/s41598-022-11421-3)
Supplement: Supplementary file 1 — Supplementary Information. [file 41598_2022_11421_MOESM1_ESM.pdf]

Daily Prosocial Actions During the COVID-19 Pandemic Contribute to Giving Behavior in Adolescence

Sophie W. Sweijen*[1], Suzanne van de Groep[1], Kayla H. Green[1],

Lysanne W. te Brinke[1], Moniek Buijzen[1], Rebecca N. H. de Leeuw[2], Eveline A. Crone[1]

[1] Erasmus School of Social and Behavioural Sciences, Erasmus University Rotterdam

[2] Radboud University Nijmegen

S1. Preregistered hypotheses

We preregistered the hypotheses tested in the current study. Here, we provide an overview of these preregistered hypotheses together with a discussion of deviations from the preregistration. The preregistration (see https://osf.io/k5xej/) and the addendum to the preregistration (see https://osf.io/yc79a/) were published on June 18, 2020, and November 25, 2020, respectively. Although data collection has begun at both timepoints, any data have not been looked at yet. While the preregistration mentions separate analyses for the two samples (i.e., high school and university students), we deviated from this preregistered analysis plan for brevity of the main manuscript.

The preregistered hypotheses on helping behavior, mood, emotional reactivity, and identity are beyond the scope of the current study. We therefore do not report in fluctuations in emotional support in this study. The results of the hypotheses on mood and emotional reactivity can be found in another study as part of this longitudinal research project[1].

Associations Between Daily Emotional Support and Giving

Preregistration

Adolescents with more daily provided emotional support are more likely to give to others (main effect daily emotional support on difference score ‘start’ and ‘end’ measurement Dictator Game targets). We expect that adolescents will give more to friends than to strangers. We expect that they will give most to people in need, which is operationalized as medical doctors, COVID-19 patients, or individuals with a poor immune system[2]. We expect that how much they give in general, and the extent to which they differentiate between targets, is positively influenced by higher levels of emotion awareness, higher levels of social reward sensitivity, stronger self-control, higher levels of general willingness to contribute to society,

better executive functioning, and greater tendencies of altruistic behavior (moderation analysis).

Deviations

We preregistered these hypotheses based on the single timepoint of data collection. Given that multiple timepoints of data collection were added after we preregistered these hypotheses, we decided to increase the power of the analyses by analyzing the raw scores of the Dictator Games (i.e., reflecting the true range of experimental giving behavior) instead of the difference score on 'start' and 'end' measurement per timepoint. This applies to all analyses on giving behavior. In addition, we decided to move the moderation analyses on emotion awareness and self-control to Supplementary S2 for brevity and clarity of the study. Finally, we exploratory examined linear and quadratic age effects on emotional support and giving behavior. Here, we also exploratory tested the effects of living situation on emotional support.

Replication analyses

Preregistration

Adolescents with more daily emotional support towards friends and family at T2 show higher giving behavior at T2, with a peak in mid adolescence. This is based on the assumption that providing opportunities for prosocial actions helps adolescents to fulfill their fundamental need of autonomy, impact and contribution to society[3,4].

Deviations

The replication analyses showed no deviations from the preregistration. However, we decided to also test whether the moderating effects of T1 could be replicated at T2. In addition, we exploratory examined linear and quadratic age effects.

Longitudinal analyses

Preregistration

Adolescents show a decrease in daily emotional support and giving behavior at T2 compared to T1[2].

Deviations

The longitudinal analyses showed no deviations from the preregistration. Data from T3 were later added to the analyses on giving behavior, but we expected to see similar patterns (i.e., decrease in giving behavior at T3 compared to T1 and T2).

S2. Additional moderation analyses

According to the preregistration, we examined the moderating effect of emotion awareness and self-control on the relation between prosocial experiences and prosocial outcomes. We report on these additional moderation analyses in the supplementary materials for brevity of the main manuscript. Based on previous studies demonstrating that emotional reactivity and self-regulation were positively associated with the exhibition of more prosocial behaviors, we tested whether emotion awareness and self-control would impact the relation between prosocial experiences and giving[5]. Here, we expected that this relation would be positively influenced by higher levels of emotion awareness and stronger self-control.

Methods

Emotion awareness

Emotion awareness was assessed with the Emotion Awareness Questionnaire – Revised (EAQ-R)[6]. Using a Likert scale ranging from 1 (*not true*) to 3 (*true*), this questionnaire measures the extent to which an individual is aware of his or her emotions. For the present study we focused on the three subscales we expected to be most associated with prosocial behaviors, namely: Attending to Others' Emotions (5 items; example item 'It is important to know how my friends are feeling.'; Cronbach's $\alpha_{\text{high school}}$ = .64 and .68 at respectively T1 and T2, and Cronbach's $\alpha_{\text{university}}$ = .58 and .66 at respectively T1 and T2), Differentiating Emotions (7 items; example item 'I never know exactly what kind of feeling I am having.'; Cronbach's $\alpha_{\text{high school}}$ = .85 and .88 at respectively T1 and T2, and Cronbach's $\alpha_{\text{university}}$ = .85 and .87 at respectively T1 and T2), and Not Hiding Emotions (5 items; example item 'When I am upset about something, I often keep it to myself.'; Cronbach's $\alpha_{\text{high school}}$ = .76 and .80 at respectively T1 and T2, and Cronbach's $\alpha_{\text{university}}$ = .84 and .83 at respectively T1 and T2). For each subscale, a mean score of the items belonging to that particular subscale at both timepoints was computed. For the scale Attending to Others' Emotions, Cronbach's α

was .64 and .68 at respectively T1 and T2 for high school students, and .58 and .66 for university students. For the scale Differentiating Emotions, Cronbach's α was .85 and .88 at respectively T1 and T2 for high school students, and .85 and .87 for university students. For the scale Not Hiding Emotions, Cronbach's α was .76 and .80 at respectively T1 and T2 for high school students, and .84 and .83 for university students.

Self-control

Self-control was measured with a one-item questionnaire that was developed specifically for the current study (see https://osf.io/h5x2a/). At both timepoints, participants were asked to rate themselves on the following statement: 'I see myself as someone who has self-control'. A 100-point scale was used, with 0 meaning 'does not describe me at all' and 100 meaning 'totally describes me'.

Results

See Table S1 for descriptive statistics and Tables S2 and S3 for correlations. Because the Mauchly's test of sphericity indicated violations of sphericity ($p < .001$), we reported all effects using the Greenhouse-Geisser correction.

Moderating effects at T1

The emotion awareness RM ANOVA with moderation analysis was performed for three subscales taken together: attending to others' emotions, differentiating emotions, and not hiding emotions. First, this analysis resulted in an interaction effect between DG target and attending to others' emotions, $F(3.28, 2020.09) = 2.53$, $p = .050$, $\eta_p^2 = .01$. Bonferroni corrected pairwise comparisons showed that attending to others' emotions was associated with giving more to friends ($B = 1.17$, $p < .001$) and individuals with a poor immune system ($B = 1.36$, $p = .035$), but not to the other targets (unknown peer, $B = -.06$, $p = .907$; medical doctor, $B = .43$, $p = .509$; a COVID-19 patient, $B = 1.38$, $p = .053$). Second, there was an interaction between DG target, emotional support to family and differentiating emotions,

F(3.28, 2020.09) = 3.11, p = .022, η_p^2 = .01. This analysis showed that for those with lower emotion awareness daily emotional support towards family was more strongly associated with giving to medical doctors (B = -.62, p = .006), but not to other targets (all ps > .05). Third, there were no effects for not hiding emotions.

No (moderating) effects of self-control were detected.

Replication analyses

The emotion awareness RM ANOVA with moderation analysis was performed for three subscales taken together: attending to others' emotions, differentiating emotions and not hiding emotions. This analysis resulted in an interaction effect between DG target and attending to others' emotions, F(3.60, 1260.57) = 2.87, p = .026, η_p^2 = .01, and an interaction effect between DG target and not hiding emotions, F(3.60, 1260.57) = 3.78, p = .006, η_p^2 = .01. Following up these interaction effects with one-way ANOVAs per subscale showed that giving to a COVID-19 patient was associated with both attending to others' emotions, F(1, 367) = 4.39, p = .037, η_p^2 = .01, and not hiding emotions, F(1, 367) = 4.69, p = .031, η_p^2 = .01. There were no effects for differentiating emotions. Whereas the findings regarding not hiding emotions and differentiating emotions are not comparable to those at T1, we replicated the interaction between DG target and attending to others' emotions.

Regarding self-control, we found an interaction effect between self-control and emotional support to friends, F(1, 356) = 4.67, p = .031, η_p^2 = .01. This result indicates that the positive relation between emotional support to friends and giving is stronger for those scoring lower on self-control. We found no such (moderating) effects of self-control at T1.

Table S1

Descriptive statistics of self-control and emotion awareness at all timepoints (T1 and T2).

Measure	# items	High School Students *N*	Min Score	Max Score	Mean	95% CI Lower Bound	95% CI Upper Bound	University Students *N*	Min Score	Max Score	Mean	95% CI Lower Bound	95% CI Upper Bound
Self-control T1	1	407	0.00	100.00	72.01	70.24	73.79	324	12.00	100.00	72.16	70.26	74.06
Self-control T2	1	211	10.00	100.00	69.53	67.08	71.98	197	0.00	100.00	70.27	67.53	73.00
Emotion awareness T1													
Attending to Others' Emotions	5	411	1.20	3.00	2.74	2.70	2.77	324	2.00	3.00	2.76	2.73	2.79
Differentiating Emotions	7	411	1.00	3.00	2.36	2.31	2.40	324	1.14	3.00	2.30	2.24	2.35
Not Hiding Emotions	5	411	1.00	3.00	1.84	1.79	1.89	324	1.00	3.00	1.96	1.90	2.02
Emotion awareness T2													
Attending to Others' Emotions	5	211	1.00	3.00	2.72	2.67	2.76	197	1.00	3.00	2.67	2.62	2.71
Differentiating Emotions	7	211	1.00	3.00	2.27	2.20	2.34	197	1.00	3.00	2.28	2.20	2.35
Not Hiding Emotions	5	211	1.00	3.00	1.78	1.72	1.85	197	1.00	3.00	1.95	1.87	2.02

Table S2

Bivariate correlations among all variables used in the statistical analyses (at T1)

	Dictator Game Day 1					Dictator Game Day 10				
	Unknown Peer	Friend	Doctor at a Hospital	COVID-19 Patient	Individual With Poor Immune System	Unknown Peer	Friend	Doctor at a Hospital	COVID-19 Patient	Individual With Poor Immune System
Attending to Others' Emotions	.129**	.215**	.112**	.179**	.138**	.094*	.155**	.092*	.102**	.110**
Differentiating Emotions	-.081*	-.100**	-.099**	-.023	-.075*	-.061	-.060	-.075*	-.040	-.032
Not Hiding Emotions	-.015	-.003	-.074*	-.003	-.013	.013	.002	-.084*	-.003	.009
Self-control	-.030	-.028	-.033	.002	-.029	-.015	-.046	-.053	-.022	-.020

Table S3

Bivariate correlations among all variables used in the statistical analyses (at T2)

	Dictator Game				
	Unknown Peer	Friend	Doctor at a Hospital	COVID-19 Patient	Individual With Poor Immune System
Attending to Others' Emotions	.069	.265**	.102*	.201**	.135**
Differentiating Emotions	-.069	.027	-.031	-.042	.008
Not Hiding Emotions	-.047	-.012	-.038	.000	.015
Self-control	-.115*	-.039	-.094	-.077	-.093

S3. Additional information on procedure

Based on the application date at T1, participants were assigned to either a batch starting on May 4, 2020 or a second batch starting on May 11, 2020. At T2, all participants started on November 9, 2020. For the follow-up session (T3), all participants completed one single questionnaire on May 24, 2021. On each testing day, participants received an invitation by email at 12:00 AM to complete a set of questionnaires. A reminder by text message was sent at 19:00 PM to the high school students and at 20:00 PM to the university students who had not yet filled in the questionnaire of that particular day. Participants were encouraged to fill in the questionnaire the same day the invitation was sent. However, they were given a longer time window to complete the questionnaire, such that high school students could fill in the questionnaires within 48 hours and the university students within 36 hours after sending the invitation. Even though different time windows were used for the two samples for convenience during data collection, most questionnaires were filled in within a 24-hour time window by both high school and university students.

S4. Replication analyses

Daily emotional support toward friends and family at T2

To examine differences in daily emotional support towards friends and family members, we performed repeated measures ANOVA with target (friends, family) as a within-subjects factor, while controlling for SES. Comparable to the findings at T1, the analysis showed a main effect of target, $F(1, 443) = 60.85, p < .001, \eta_p^2 = .12$, such that adolescents showed more emotional support towards friends (*M* = 2.70, 95% CI [2.50, 2.90]) than family (*M* = 2.25, 95% CI [2.02, 2.48]). The RM ANOVA also resulted in an interaction effect between target and linear age, $F(1, 443) = 41.29, p < .001, \eta_p^2 = .09$. Whereas no significant age effects were found on emotional support to family, emotional support to friends decreased linearly with age, $F(1, 457) = 20.57, p < .001, \eta_p^2 = .04$. There were no main and interaction effects with gender.

Giving toward unfamiliar peers, friends, and COVID-19 targets at T2

To examine giving behavior toward different targets, we performed a repeated measures ANOVA with DG target (unknown peer, friend, medical doctor, COVID-19 patient, and individual with a poor immune system) as within-subjects factor. SES was added as control variable. The analysis resulted in a main effect of target, $F(3.62, 1587.46) = 51.99, p < .001, \eta_p^2 = .11$. Post-hoc pairwise Bonferroni corrected comparisons revealed all targets differed significantly from each other (*p*'s < .001), except for given coins to a friend did not differ a COVID-19 patient and an individual with a poor immune system. Most coins were given to a medical doctor (*M* = 5.96, 95% CI [5.50, 6.41]). Fewer coins were donated to an individual with a poor immune system (*M* = 5.18, 95% CI [4.75, 5.62]) and a COVID-19 patient (*M* = 5.12, 95% CI [4.67, 5.58]), followed by a friend, (*M* = 4.95, 95% CI [4.70,

5.20]). The least number of coins were given to an unknown peer, (M = 3.09, 95% CI [2.73, 3.44]). These findings are comparable with those at T1. The RM ANOVA further resulted in a main effect of linear age, $F(1, 438) = 4.56$, $p = .033$, $\eta_p^2 = .01$, indicating that giving behavior decreased with age, as well as an interaction effect between target and quadratic age, $F(3.62, 1587.46) = 2.86$, $p = .027$, $\eta_p^2 = .01$. This interaction effect was driven by giving behavior towards a doctor (B = -.07, p = .034) and a COVID-19 patient (B = -.08, p = .022), such that giving to these deserving targets decreased with age. Consistent with the findings at T1, the analysis also yielded an interaction effect between target and gender, $F(3.62, 1587.46) = 3.95$, $p = .005$, $\eta_p^2 = .01$. Females showed higher giving behavior toward the COVID-19 related targets compared to males (all p's < .05).

Associations between daily emotional support and giving at T2

To examine the effect of daily emotional support on giving behavior, a repeated measures ANOVA was conducted with DG target as within-subject factor and emotional support to each target (friends, family) as covariate, controlling for linear age, gender, and SES. The analysis showed a main effect of emotional support to friends, $F(1, 424) = 5.44$, $p = .020$, $\eta_p^2 = .01$, indicating that adolescents with higher levels of emotional support to friends gave more coins to others. The analysis also yielded an interaction between target and emotional support to family, $F(3.64, 1542.71) = 3.06$, $p = .019$, $\eta_p^2 = .01$. Emotional support to family was significantly associated with giving to a COVID-19 patient (B = .29, p = .042), but no such effects were found for the other targets (all p's > .05). These effects are highly comparable to those at T1.

Emotional and cognitive factors – moderators at T2

Finally, we examined moderating effects of emotional and cognitive factors on the association between daily emotional support and giving behavior. For each moderator, we

performed a separate repeated measures ANOVA with DG target as within-subject factor, and emotional support to friends or family and the moderator as predictors (allowing for interactions between emotional support and the moderator). We controlled for gender, SES and age by adding these as between-subjects factors and covariates, respectively. Given that SRQ and altruism were only administered at T1, these moderation effects were not replicated at T2.

Executive functions

Regarding executive functioning, the analysis showed a main effect of executive functioning, $F(1, 356) = 6.78$, $p = .010$, $\eta_p^2 = .02$, and an interaction effect between executive functioning and emotional support to friends, $F(1, 356) = 6.86$, $p = .009$, $\eta_p^2 = .02$. These results indicate that the positive relation between emotional support to friends and giving is stronger for those scoring higher on executive functioning. We found no such (moderating) effects of executive functioning at T1.

GCS

We observed a main effect of GCS on giving, $F(1, 356) = 11.10$, $p = .001$, $\eta_p^2 = .03$, such that adolescents who are generally more willing to contribute to society are more likely to give to others. Additionally, the analysis yielded an interaction effect between target and GCS, $F(3.62, 1286.89) = 2.65$, $p = .037$, $\eta_p^2 = .01$. Bonferroni corrected pairwise comparisons showed that societal awareness was associated with giving to an unknown peer ($B = .30$, $p = .045$), a friend ($B = .38$, $p < .001$), a COVID-19 patient ($B = .69$, $p < .001$) and an individual with a poor immune system ($B = .51$, $p = .004$), but not to a medical doctor ($p > .05$).

S5. Additional emotional support analyses

Given significant correlations between the two emotional support items as covariates in the repeated measures ANOVA examining the association between emotional support to friends and family and giving behavior (p < .05), additional analyses were performed using the average score of the two items as a single covariate. To examine the effect of daily emotional support on giving behavior at the first timepoint (T1), a repeated measures ANOVA was conducted with DG time and DG target as within-subject factors and with emotional support as covariate, controlling for gender, age and SES. The analysis showed highly similar results to the analyses with the two targets as separate covariates. That is, the analyses showed a main effect of emotional support, $F(1, 692) = 18.21, p < .001, \eta_p^2 = .03$, such that adolescents with higher levels of daily emotional support donated more coins to others. In addition, we found an interaction between emotional support and target, $F(3.31, 2293.28) = 8.19, p < .001, \eta_p^2 = .01$. Emotional support was significantly associated with giving to friend ($B = .17, p < .001$), doctor in hospital ($B = .38, p < .001$), COVID-19 patient ($B = .44, p < .001$), and individual with a poor immune system ($B = .47, p < .001$). Whereas the interaction effect could not be replicated at T2, we did replicate the main effect of emotional support on giving, $F(1, 438) = 17.37, p < .001, \eta_p^2 = .04$.

References

1. Green, K. H. *et al.* Mood and emotional reactivity of adolescents during the COVID-19 pandemic: short-term and long-term effects and the impact of social and socioeconomic stressors. *Sci. Rep.* **11**, 1–13 (2021).
2. van de Groep, S., Zanolie, K., Green, K., Sweijen, S. W. & Crone, E. A. A daily diary study on adolescents' mood, empathy, and prosocial behavior during the COVID-19 pandemic. *PLoS One* **15**, (2020).
3. Yeager, D. S., Dahl, R. E. & Dweck, C. S. Why interventions to influence adolescent behavior often fail but could succeed. *Perspect. Psychol. Sci.* **13**, 101–122 (2018).
4. Fuligni, A. J. The need to contribute during adolescence. *Perspect. Psychol. Sci.* **14**, 331–343 (2019).
5. Carlo, G., Crockett, L. J., Wolff, J. M. & Beal, S. J. The role of emotional reactivity, self-regulation, and puberty in adolescents' prosocial behaviors. *Rev. Soc. Dev.* **21**, 667–685 (2012).
6. Rieffe, C., Oosterveld, P., Miers, A. C., Meerum Terwogt, M. & Ly, V. Emotion awareness and internalising symptoms in children and adolescents: The Emotion Awareness Questionnaire revised. *Pers. Individid. Dif.* **45**, 756–761 (2008).
